# A Review of Rare Disease Policies and Orphan Drug Reimbursement Systems in 12 Eurasian Countries

**DOI:** 10.3389/fpubh.2019.00416

**Published:** 2020-01-28

**Authors:** Marcin Czech, Aleksandra Baran-Kooiker, Kagan Atikeler, Maria Demirtshyan, Kamilla Gaitova, Malwina Holownia-Voloskova, Adina Turcu-Stiolica, Coen Kooiker, Oresta Piniazhko, Natella Konstandyan, Olha Zalis'ka, Jolanta Sykut-Cegielska

**Affiliations:** ^1^Department of Pharmacoeconomics, The Institute of Mother and Child, Warsaw, Poland; ^2^Department of Pharmacoeconomics, Faculty of Pharmacy, Medical University of Warsaw, Warsaw, Poland; ^3^Division of Pharmacoepidemiology and Clinical Pharmacology, Faculty of Science, Utrecht Institute for Pharmaceutical Sciences, Utrecht, Netherlands; ^4^Unit of Health Technology Assessments, Turkish Ministry of Health, Turkish Medicines and Medical Devices Agency, Ankara, Turkey; ^5^Ascent Global Market Solutions (Non-profit), Walnut Creek, CA, United States; ^6^Center for Economics and Health Technology Assessment, Republican Center for Health Development, Ministry of Health, Nur-Sultan, Kazakhstan; ^7^State Budgetary Institution Research Institute for Healthcare Organization and Medical Management of Moscow Healthcare Department, Moscow, Russia; ^8^Department of Experimental and Clinical Pharmacology, Medical University of Warsaw, Warsaw, Poland; ^9^Department of Pharmacoeconomics, Faculty of Pharmacy, University of Medicine and Pharmacy of Craiova, Craiova, Romania; ^10^Independent Researcher, Warsaw, Poland; ^11^Department of Management and Economy of Pharmacy, Medicine Technology and Pharmacoeconomics, Postgraduate Faculty, Danylo Halytsky Lviv National Medical University, Lviv, Ukraine; ^12^Republican Center of Medical Genetics, Yerevan State Medical University, Yerevan, Armenia; ^13^Department of Inborn Errors of Metabolism and Paediatrics, The Institute of Mother and Child, Warsaw, Poland

**Keywords:** rare diseases, newborn screening, national plan, patient registries, reimbursement, policy

## Abstract

**Background:** Despite international initiatives on collaboration within the field of rare diseases, patient access to orphan medicinal products (OMPs) and healthcare services differ greatly between countries. This study aimed to create a comprehensive and in-depth overview of rare diseases policies and reimbursement of OMPs in a selection of 12 countries in the Western Eurasian region: Armenia, France, Germany, Kazakhstan, Latvia, The Netherlands, Poland, Romania, Russia, Turkey, Ukraine, and the United Kingdom.

**Methods:** A systematic literature review was performed and an analysis of publicly available legislative and rare disease health policy data was undertaken in five focus areas: rare disease definition, newborn screening, registries, national plans, access to/reimbursement of OMPs.

**Results:** Screening programs are broadly implemented but the number of screened diseases differs significantly (2–35 diseases), either between EU and non-EU countries, between EU member states and sometimes even within a single country. In most countries rare disease registries are operating with regional, national, European or worldwide coverage. The number of rare disease registries is growing, as a result of the National Plans (EU) and increased international scientific cooperation. France, Russia, and Poland have a centrally acting registry. National plans are present in all EU countries but implementation varies and is ongoing. The number of reimbursed OMPs in the selected countries ranges from nearly all available OMPs in the Netherlands, Germany, and France to zero in Armenia. Reimbursement rules differ considerably regionally and a trend is observed of reimbursement conditions getting stricter for expensive (orphan) drugs.

**Discussion:** Inequality in patient access to new OMPs still exists due to variations in national policies, healthcare budgets, health insurance, and reimbursement systems. The observed differences are challenging for rare disease patients, health authorities and manufacturers alike. Progress can be seen, however, and international cooperation and harmonization is slowly but steadily expanding in the rare disease arena.

## Introduction

Between 6,000 and 8,000 rare diseases have been identified, most of genetic origin and with severe clinical manifestations. Due to insufficient knowledge on disease pathology, diagnosis is frequently delayed, often resulting in early and irreversible complications. Thirty percent of rare disease patients die before the age of five^1^. Pharmacotherapy, known as orphan drugs or Orphan Medicinal Products (OMPs), exists for <3% of rare diseases^2^ (1–3). Registration and reimbursement are the two main policy hurdles before a drug can reach a patient. Regulatory legislation for OMPs has been harmonized across the European Union (EU), with simultaneous regulatory approval for OMPs across 28 member states (4). However, differences remain in reimbursement and pricing systems in member states, based on factors such as healthcare budget (related to a country's GDP), type of healthcare and health insurance system, patient co-payment rules, reimbursement timelines and evidence requirements (i.e., type, level, and presentation). Consequently, patient access is often unpredictable and restricted while reimbursement strategies for manufacturers are fragmented and complex. The high prices of many orphan drugs, often combined with a limited amount of clinical evidence (mainly due to small patient populations), can lead to Incremental Cost-Effectiveness Ratios (ICER) that exceed “willingness to pay” levels (5). Budget restriction measures, especially around “expensive drugs” (which OMPs often are), are increasingly common. Reference pricing methods (i.e., HTA agencies comparing and referencing to drug prices in other countries or regions) can influence manufacturers to postpone or even avoid entering certain markets due to a possible cascading price-drop effect elsewhere (6). These are a few of the factors that can cause inequality in patient access to new medical technologies and treatments (7). A 2017 survey by EURORDIS confirmed that 24% of rare disease patients did not receive treatment because of no drug availability in their country (vs. 7% of the general population) and 15% due to inability to pay for treatment (vs. 6%) (8).

A recent step toward HTA harmonization between EU member states is the official proposal of the EU Health Technology Assessment (HTA) Regulation in 2018, which has been planned to be adopted in 2019. A pivotal component of this regulation is a centralized Joint Clinical Assessment (JCA) at the European level, which is aimed at establishing the (clinical) value of the treatment for HTA purposes (9). Such a central assessment would reduce HTA workload in the individual member states, promote the sharing of knowledge and leverage the expertise of rare disease experts and patient representatives in the EU. In essence, the JCA resembles the shared regulatory assessment done by the European Medicines Agency (EMA) in the Centralized Procedure (9). The JCA could improve the quality and speed of HTA for OMPs at the national level and promote further HTA harmonization. However, details on implementation, member state representation and how the joint clinical assessment will be legally binding (for national HTA purposes) is still under discussion and some concerns are already being voiced (10). The final HTA decision making, which depends on country specific factors such as the structure of the healthcare system, reimbursement factors and budgeting aspects, will likely remain at the national level.

Rare disease policies are a high focus area, given the medical need surrounding rare diseases and the relatively large impact these diseases and their treatment potentially have on healthcare budgets. The reimbursement status of orphan drugs in Eastern Europe has been described by several authors recently (11–15). There have been multiple publications describing OMP policies in Central and Eastern Europe in single countries (16–18) or covering a larger number of countries in Europe (5, 19–22). Pejcic et al. focused on HTA and pricing as well as rare disease policies in 14 Eastern European countries (23). In 2015 Gammie et al. presented a comprehensive review of legislations, regulations and policies in 35 countries describing in detail the national orphan drug policies, orphan drug marketing authorization processes (and accelerated procedures), incentives, marketing exclusivity, pricing, and reimbursement (2015) (24). Dharssi et al. evaluated key patient-needs across five dimensions: improving coordination of care, diagnostic resources, access to treatment, patient awareness and support, and promoting innovative research in 11 EU and non-EU countries (25).

However, there is still little comprehensive and in-depth information available in the English literature on orphan drug policies and HTA processes within the European Commonwealth of Independent States (CIS), such as in Russia, Armenia, and Kazakhstan in comparison to European Union countries. This field is rapidly evolving due to implementation of national plans for rare diseases in some European countries and HTA developments. Therefore, the aim of this article is to bridge the identified gaps by presenting an overview and comparison of current rare disease policies, HTA and reimbursement processes for orphan drugs in a broader range of Eurasian countries.

## Materials and Methods

For this publication an analysis of rare disease policies was undertaken, focused on the following topics, including several “core areas” as defined by the EU (Council Recommendations of 2009) (26):

- Rare disease definition,
- Newborn screening (NBS) for rare diseases,
- National plans (NP) for rare diseases,
- Rare disease registries (central vs. disease-specific),
- Reimbursement and HTA approaches for orphan drugs, including access to orphan drugs (measured by the number of reimbursed OMPs) and availability of early access methods (e.g., compassionate use, named patient-programs, conditional reimbursement).

Other aspects mentioned in the 2009 EU Council Recommendation such as research on rare diseases empowerment of patient organizations, and sustainability were not researched as they are difficult to quantify and assess in an objective manner. Codification and inventorying of rare diseases were excluded as well in this paper, as these have little direct impact on treatment. In addition, the authors decided to include newborn screening, reimbursement (incl. early access programmes) and HTA processes, in order to present a more holistic overview of rare disease policies in each country.

The 12 countries included in this study were selected to be diverse from a geographical and socio-economical viewpoint and represent a wide range of rare disease policy development across the western Eurasian region: Armenia (AM), France (FR), Germany (DE), Kazakhstan (KZ), Latvia (LV), The Netherlands (NL), Poland (PL), Romania (RO), Russia (RU), Turkey (TR), Ukraine (UA), and the United Kingdom (UK).

**Figure 1:**
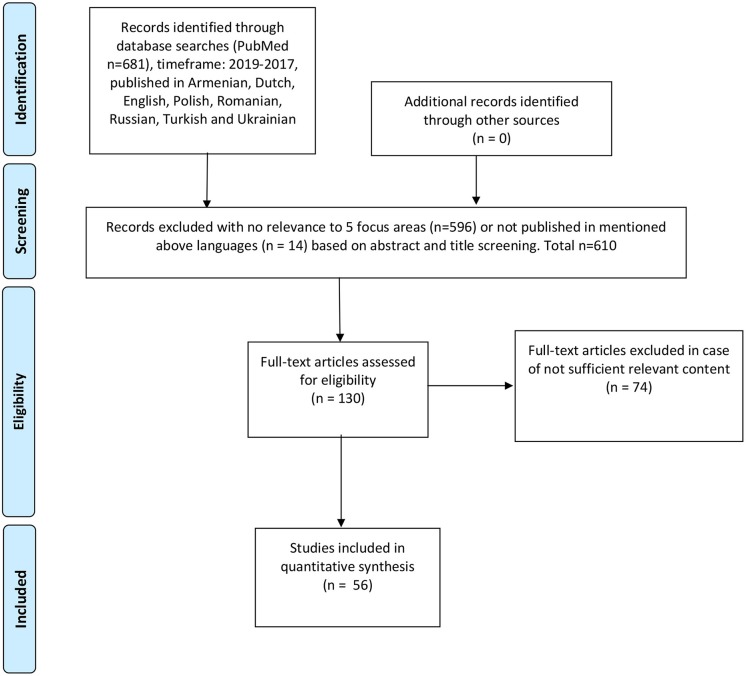
Reproduced with permission from PRISMA 2009 flow diagram (27).

A systematic literature review was performed to identify previous research and relevant publications, using the following keywords: rare disease, rare disorder, orphan drug, orphan medicinal product, health policy, reimbursement, HTA, health technology assessment, newborn screening, patient registry, national plan, legislation, access, Poland, Germany, Netherlands, Holland, Kazakhstan, Russia, Ukraine, Turkey, Armenia, France, UK, United Kingdom, England, Scotland, Northern Ireland, Wales, Romania, Latvia. Articles published from 2017 to 2019 were included. The review resulted in 681 publications that were screened by title/abstract, 610 publications were excluded due to insufficient relevance to the selected focus areas, 71 full-text articles were assessed for eligibility, of which 10 were included. All steps of the literature review (identification, screening, eligibility, inclusion, and data extraction) were performed by two independent researchers, according to PRISMA methodology (please refer to Figure 1).

In order to gather further information in the scope of the article, an explorative internet search (gray literature review) was done of publicly listed policies, legislations, guidelines, governmental publications and other sources of relevant orphan drug HTA information. This was done by searching the websites of local Health Authorities, e.g., the Ministry of Health and HTA agency. The most up-to-date data the authors could find was included. Experts from all countries were interviewed to confirm the obtained information or in case public information was insufficient, unclear, contradictory or lacking. The authors' intention was to select a fair representation of different types of stakeholders involved in market access processes of orphan drugs. Public institution representatives, payers, scientists, clinicians, and commercial entity representatives from the countries were interviewed. Their number was dependent on the quality of information available from public sources and the willingness of stakeholders to provide additional data as well as a degree of involvement in the study. A list of questions was sent to the experts by email and followed up by phone interviews. Approval by an ethics committee was not required for this research.

### Definition of Rare Disorders, Orphan Drugs and Epidemiology

The EU has officially defined rare diseases as being rare when they affect fewer than 1 in 2000 (i.e., a prevalence of 5 or less per 10,000) (28) and in most of the selected countries this definition is used [FR, DE, LV, NL, PL, RO, UK, and UA (29, 30)]. In Russia the maximum prevalence for a rare disease is defined as 1 in 10,000 (31). There is no data available on the maximum prevalence for a rare disease in Kazakhstan (32). Some countries use additional definitions in situations where a condition is not officially defined as rare, such as in the UK, where the National Health Service (NHS) classifies all conditions that require specialized medical care also as rare if they occur in <500 citizens yearly (29)^3^. Turkey defines a rare disease when they affect no more than 1 in 100,000, which is 50 times less frequent than the European Union definition (33, 34). There is no specific definition for “rare disease” in Armenian legislation, only “levels of disability” which define whether the patient will receive the necessary medicines for free or not^4^.

The Netherlands defines the classification “orphan drug” as either having an official EU orphan designation or if it targets a disease with a prevalence of <1 in 150,000 and shows a clinically proven therapeutic benefit and no other registered medicine exists^5^.

France introduced an extra definition of “rare cancer” if the cancer occurs in <6 in 100,000 per year or requires specialized treatment due to untypical tumor location or complex disease characteristics (29, 35). Effective from October 2018, Scotland has introduced a new definition for ultra-orphan drugs: “medicines that are used to treat a condition with a prevalence of 1 in 50,000 or less or around 100 people in Scotland,” which will mostly be used to facilitate early access programs and reimbursement processes^6^.

### Newborn Screening

Newborn screening (NBS) is used to identify and effectively treat certain rare disorders at an early stage and to prevent irreversible damage. NBS is performed in all countries selected for this review. There is, however, a lack of uniformity between screening programs, mainly in the number of screened disorders, ranging from 2 to 35 (see Table 1). Poland currently screens for 28 rare diseases (36), The Netherlands 20 (37, 38), Germany 15^7^^,^^8^ (39), France and Russia 5 and (N.B.: 35 in Moscow) (29)^9^, Ukraine 4 (41), Turkey 6 (40, 42)^10^. Armenia^4^, Kazakhstan^11^ (43), Latvia and Romania^12^ (44, 45), only screen for phenylketonuria and congenital hypothyroidism. England, Scotland, and Wales screen for nine diseases, whereas Northern Ireland (part of the UK as well) screens only for 5 (29)^13^^,^^14^. In several countries the number of screened diseases is being expanded or planned to be expanded, notably in Turkey (going from 6 to 10 screened diseases)^11^ and the Netherlands (from 20 to 32) (37), but without specific timelines.

**Table 1:** New born screening of rare diseases per country (29)^4^, (36–38)^7^^,^^8^, (39)^9^, (40–42)^10^^,^^11^, (43)^12^, (44, 45)^13^^,^^14^.

	**AR** **(4)**	**DE** **(15)**	**FR** **(5)**	**KZ** **(2)**	**LV** **(2)**	**NL** **(20)**	**PL** **(28)**	**RO** **(2)**	**RU** **(5)**	**TR** **(6)**	**UA** **(4)**	**UK:** **NI** **(5)**	**UK:** **ENG/WAL/SCO** **(9)**
Argininemia							X						
Argininosuccinic aciduria (ASA)							X						
Alfa –Thalassemia/HbH disease						X				P			
Beta-Thalassemia						X							
Beta-ketothiolase deficiency						P	X						
Biotinidase deficiency (BIO)		X				X	X			X			
Carnitine-acylcarnitine translocase deficiency (CACT)		X				P	X						
Carnitine transporter deficiency (OCTN2)						X	X						
Carnitine palmitoyltransferase deficiency type I & II (CPT-1, CPT-2)		X				P	X						
Congenital adrenal hyperplasia (CAH) or Adrenogenital syndrome (AGS)		X	X			X	X		X		X		
Congenital hypothyroidism (CH)	X	X	X	X	X	X	X	X	X	X	X	X	X
Cystic fibrosis (CF)		X	X			X	X		X	X	X	X	X
Citrullinemia type I & II							X						
Developmental hip dysplasia	X									X			
Galactosemia (GAL)		X				X			X	X			
Galactokinase deficiency (GALK)						P							
Guanidinoacetate methyltransferase deficiency (GAMT)						P							
Glutaric acidemia type 1 (GA-1)		X				X	X						X
Glutaric acidemia type 2							X						
HMG-CoA-lyase deficiency (HMG)						X	X						
Homocystinuria (HCU)							X						X
Isovaleric acidemia		X				X	X						X
Long Chain 3-hydroxyacyl-CoA Dehydrogenase Deficiency (LCHADD)		X				X	X						
Maple syrup urine disease (MSUD)		X				X	X						X
Medium Chain Acyl-CoA Dehydrogenase Deficiency (MCADD)		X				X	X					X	X
3-Methylcrotonyl-CoA carboxylase deficiency (3-MCC) (3-methylcrotonylglycinuria)						X	X						
Methylmalonic academia (MMA)						P	X						
Mitochondrial trifunctional protein deficiency							X						
Mucopolysaccharidosis type 1 (MPS I)						P							
Multiple CoA carboxylase deficiency (MCD)						X	X						
Phenylketonuria/hyperphenylalaninemia (PKU)	X	X	X	X	X	X	X	X	X	X	X	X	X
Propionic acidemia (PA)						P	X						
Retinopathy of prematurity	X												
Severe combined immune deficiency (SCID)						P							
Sickle Cell Disorder (bearer)		P	X			X						X	X
Tyrosinemia type 1 (TYR-1)		X				X	X						
Tyrosinemia type 2 (TYR-2)							X						
Very Long-Chain Acyl-CoA Dehydrogenase Deficiency (VLCADD)		X				X	X						
X-linked adrenoleukodystrophy (X-ALD)						P							

*X, newborn screening performed; P, Pilot or planned to be extended (newborn hearing test not included)*.

### National Plans for Rare Diseases

In 2009 the European Council issued the recommendation for EU member states to create and adopt a plan focused on rare disorders by the end of 2013, with the goal to have an overall Community strategy for “ensuring effective and efficient recognition, prevention, diagnosis, treatment, care, and research for rare diseases in Europe” (46). For this purpose, the European Project for Rare Diseases National Plans Development (EUROPLAN) was introduced to promote and help EU members with the construction and implementation of their national plans^15^^,^^16^.

NL, DE, UK, LV have created a national plan within the timelines defined by European Commission but in most of these EU countries the implementation is in progress (16, 29)^17^^,^^18^^,^^19^^,^^20^. In Poland and Romania, a National Plan for RDs was developed but has never been implemented^21^^,^^22^. The most recent version of Polish Plan for RDs for 2017–2019, was written under the auspices of the Polish MoH and was planned to be approved in the 3rd quarter of 2019^22^^,^^23^. France was a forerunner in introducing a National Plan in 2004, with an assigned budget of €100 M for implementation over 2005–2008 (29). The 3rd French national plan has been created for 2018–2022. Rare disorder patients in France can also receive support from the so-called Cancer Plan (latest version 2014–2019, in case of rare oncological diseases, and the National Plan for Rare Handicaps (2014–2018), addressing rare physical disabilities (29, 47–49).

In Russia a special program exists (on the federal level) for financing 12 high-cost diseases: hemophilia, cystic fibrosis, pituitary dwarfism, Gaucher disease, lymphoid malignant neoplasms, hematopoietic and related tissues, multiple sclerosis, hemolytic-uremic syndrome, juvenile arthritis with systemic onset, mucopolysaccharidosis type I, II, and VI^24^ (50).

Both Kazakhstan and Turkey have national programmes for rare diseases, but they are undergoing implementation. In the non-EU countries in this review (KZ, TR) (40, 51–53) a national strategy targeting rare diseases was not adopted and, in some cases there is even a complete lack of legislation that addresses the needs of rare disease patients and orphan drug topics (e.g., AM)^4^. Table 2 describes the most important characteristics of the national plans and their status at the time of writing.

**Table 2:** Description of National Plan for Rare Diseases per country (16, 29)^4^, (54–64)^25^^,^^26^, (65)^27^, (66)^28^^,^^29^.

**Country**	**Characteristics of the national plans**
AR	No NP or special legislation for rare diseases.
DE	NP developed and implemented: 7 focus areas, 52 proposed solutions, implemented in 2013. Twenty-eight rare disease institutions have been working together under the name NAMSE since 2009. In 2015 an online information portal (project ZIPSE) has been created, and an interactive map for patients to find centers of expertise.
FR	NP developed and implemented. 1st Ed. 2005–2008: 10 priority areas, budget €100 M. 2nd Ed.: 2011–2014 (budget €180 M), extended to 2016. Focus areas: improve quality of care for RD patients, more international collaboration and French research. Forty-seven specific steps for plan realization, incl. an audit. 3rd Ed. Cancer Plan 2014–2019 (incl. rare cancers). Definition of rare cancer introduced <6/100,000 per year or specialized treatment required due to atypical tumor location or cancer complexity. 2nd edition Cancer Plan structured functions among cancer centers. The NP for “Rare Handicaps” 2009–2013 was created by CNSA (National Solidarity Fund for Autonomy), which financially supports elderly frail and disabled people. The plan focuses on improving access to information on rare disabilities, having unified diagnostic and disease qualification processes, reference centers and introducing specialized care for rare disabilities. The 2nd Edition (2014–2018) has four priorities: support societal integration processes, improve quality of life, ensure age-independent medical care, and support clinical trials. CNSA is responsible for implementation.
KAZ	No NP.
LV	NP developed and implemented. Created for 2013–2015 by a working group consisting of representatives of HCPs, MoH, and patients organizations. The NP was accepted in 2013 but without any budget. Main priorities: access to information on rare diseases and registry creation. Due to lack of resources, the NP only has an organizational and structural role, but not practical.
NL	NP developed and implemented (NPZZ), but has not come to full fruition yet (2017). The plan contains observed hurdles (awareness, organization, research, role of patient organizations, need for coordination), several recommendations (education of HCP's, information management, healthcare organization and access to treatment, scientific research, appointment of a RD coordinator), and both short and long-term priorities within these areas The ZonMW institute has reviewed development/implementation of the NP since 2015, to structure and prioritize the multitude of observations and recommendations. The final recommendation to the MoH was given by ZonMW in February in 2017, with a large focus on creating 300 reference centers (completed) and their role in coordinating healthcare access and expertise down to local healthcare providers.
PL	First draft of an NP was developed by the National Forum for Rare Disorders with the Team for Rare Disorders in 2012, but not implemented (2017). The draft describes in detail screening, diagnostic and genetic tests, reference centers, multidisciplinary care, integrated social support systems for patients and families, education on rare diseases, sources of information, access to orphan drugs and a central registry of rare disorders. A new Plan for Rare Diseases was created in 2017 under leadership of the Polish Ministry of Health with an intention to be implemented in the near future.
RU	No NP.
RO	The MoH, National House for Health Insurance with the National Alliance for Rare Diseases (RONARD) started working on the NP in 2008 and it was proposed in the National Health Program. The draft of Romanian national plan was never adopted as a separate policy document with an allocated budget. Eight priorities were emphasized in this plan: - Establishing legal, social, economic norms and principles - Developing a network/chain of centers involved in diagnosis, treatment, rehabilitation and prevention - Facilitating access to the newest medicinal products and technologies - Improving access to information on rare diseases - Educating doctors - Involvement in clinical trials on rare disorders - Empowerment of patients' organizations and strengthening their role - Development of cooperation with other European countries. The MoH appointed the National Council for Rare Diseases, which has a consultative role and coordinates the implementation of the NP. Due to economic reasons, the implementation period has been extended to 2020.
UA	No NP exists, but legislative amendments concerning rare diseases have been introduced in 2014 (approved in 2015), via which the official list of rare diseases (256 diseases) has been published and rules for reimbursement of OMP's (by state and local budget) were defined and disease registries were introduced. In addition, the “National Action Plan to implement the UN Convention on the Rights of the Child” (August 2016) includes prioritization of pediatric rare disorders.
UK	NP developed and implemented. The National Strategy for rare diseases has been accepted by the MoH in 2013, incorporating 51 commitments for patients with rare disorders to be fulfilled by 2020. Commitments are broad and concern diagnostics, access to information, improvement of healthcare, creation of disease registries, clinical trials. Implementation has started in regions and progress is monitored.
TR	No NP.

### Disease Registries

A limited number of registries for rare disorders exist in most of the selected countries, even though it is a focus topic in many of the national plans. The first outcomes of implementing rare disease registries are already visible and resulted in scientific collaboration such as the Network dedicated to Rare Adult Cancer (RAC), through which knowledge on epidemiology, survival prognosis, prevalence, burden of rare cancers is shared. Registries are either public or private non-profit or for profit (54–56)^25^.

France implemented a central registry (fr. Banque Nationale de Données Maladies Rares, BNDMR) that collects data for all rare disorders, next to 12 other rare disorder registries. The central registry gathers epidemiological data in order to optimize clinical practice and healthcare policies. It also serves to facilitate patients to therapeutic programs and clinical trials. Rather uniquely, data on patients' family members is also collected. The epidemiological data is aggregated within the centers for rare diseases (Centres des maladies rares) CEMARA program (replaced by the BaMaRa application in 2017) which has identified more than 380,000 patients and 4,200 rare disorders (29, 57–59). Since 2017 109 CRMRs (multi-site reference centers) were created, 387 reference centers and 1,757 competence centers identified, as well as 83 resource and competence centers (CRCs) (67). Up to May 2019 there were 143 RD registries in France (60). Poland has a Central Registry for Inherited Disorders which is obligatory to report birth defects to since 2014 as well as 10 disease specific registries^26^.

Germany has acted on the NP recommendation to create disease registries such as the Open Source Registry System for Rare Diseases (Open-Source-Registersystem für Seltene Erkrankungen) (29, 61)^25^. Currently in Germany there are 149 RD registries (13 regional, 94 national, 18 European, and 24 global)^25^ and a central portal (61, 62).

The UK has 74 functioning registries under control (incl. 12 global, 13 European), also by public or private institutions (29)^25^.

Latvia has one registry for multiple diseases, called the “Registry for Certain Diseases,” which include rare cancers, hereditary disorders, managed by the Centre for Disease Prevention and Control. There are plans to implement a central registry for rare disorders within the national plan (16, 29)^25^.

Until May 2019 32 RD registries in The Netherlands existed, however, the national plan led to appointing around 350 reference centers that are able to comply with the EU standards, including 5 of the 24 new European Reference Networks (ERN) (65)^27^. The large number of centers will be working together in clusters, to prevent fragmentation (66).

Romania has two disease registries (biliary atresia and cystic fibrosis), both contributing to European registries^28^.

Turkey has five working registries, one for oral ulcers in Behcet disease, cystic fibrosis (contributing to EUROCARE cystic fibrosis registry), Duchenne, Becker, and spinal muscular dystrophy (contributing to TREAT-NMD), pediatric atypical hemolytic uremic syndrome, severe chronic neutropenia (contributing the SCN international registry) (40, 63). A registry for rare pediatric metabolic disorders is financed by Hacettepe University Hospital and the Metabolic Disease Foundation (METVAK). Turkey participates in European registries E-IMD (40, 64).

Russia is the only non-EU country in this review having a central rare disorder registry (31). There are no official rare disease registries in Kazakhstan, but work is underway to establish a national rare disease registry to help identify common genetic mutations within the Kazakh population, which is intended to collaborate internationally (51)^29^. Armenia has no registries^4^ and is also the only country that does not have patient organizations gathering data. Disease registries are under development in Ukraine, which currently has one, for spinal muscular atrophy^24^.

### Rare Disease Policies and Access to Orphan Drugs

Although the European Commission has granted 2121 “Orphan Designations” from 2000 until 2019, “only” 164 orphan drug marketing applications were approved via EMA's centralized procedure in this period (1–3).

In contrast to the regulatory process, which is performed centrally and leads to a simultaneous drug approval for all 28 EU members, health technology assessment, pricing, and reimbursement are still executed on the national level. This can lead to differences in patient access, as illustrated below. Data from 2015 shows that the Netherlands reimbursed all OMPs registered in the EU except 3 (Ceplene®, Mepact®, and Bronchitol®) (68). In Germany the total number of reimbursed OMPs was 133^30^. Since the 2011 introduction of legislation aiming at controlling prices of patented pharmaceuticals and to curb spending (Act to Reorganize the Pharmaceuticals' Market in the Statutory Health Insurance System, AMNOG) until March 1st 2017, 51 orphan drug reimbursement procedures have been finalized by Germany's Federal Joint Committee (Gemeinsame Bundesausschuss, G-BA)^31^. OMPs are most widely accessible in Germany and France (69).

France reimburses 116 orphan drugs, England 68, Scotland 55, and Wales 47 (65). England, <50% of centrally authorized OMPs are routinely funded by the NHS, with one-third of these recommended by NICE (69).

Latvia reimburses 25 orphan drugs, 21 via three reimbursement pathways (the reimbursement list, individual reimbursement and the CCUH program “Medicinal treatment for children with rare diseases”) and 4 through multiple reimbursement mechanisms (15).

Poland reimburses 48, the vast majority of which within so-called “Drug Programs” (DPs), introduced by the MoH in 2012 for expensive medical technologies (replacing previous “therapeutical programs”) (11, 70–72). DPs are mainly designed to control consumption of the most expensive drugs^22^.

Romania has 70 reimbursed OMPs^21^^,^^32^. Russia has been reimbursing 27 high-cost drugs for orphan diseases on the federal level and 43 in the Moscow region (73–75)^33^, which is an example of regional differences in patient access. Ukraine reimburses 23 active substances for 7 diseases approved for state procurement based on the national drug program inclusion criteria (76, 77), 12 diseases for children and adults, covering 65 INNSs.

In Turkey currently 43 orphan drugs are reimbursed but 22 of them are not currently marketed in Turkey, for this reason, Social Security Institutions use direct importation for those products (78, 79).

Kazakhstan has 42 reimbursed OMPs at the country level and 2 reimbursed rare disease funds. However, according to the Kazakh definition of orphan drugs/rare diseases there are 150 orphan drugs for 50 disease classes (80, 81).

In Armenia there is no reimbursement as seen in the other countries: many medicines are given via donations^4^. Medicines are distributed free of charge from the MoH warehouse to polyclinics and hospitals nationwide. All medicines are obtained through tenders posted by the Armenian MoH. When a rare disease does not cause physical or mental disability, all costs for required medicines or medical nutrition are borne by the patient^4^.

### Early Access (Compassionate Use, Named Patient Programme, Conditional Reimbursement)

According to Balasubramanian et al., 20 out of 28 EU member states had an established compassionate use programme (CUP) (82). A CUP exists in all EU countries selected for this review, except Poland (work on implementation of a national CUP is ongoing)^23^ (82).

In the EU it is also possible to request a CUP centrally via the EMA Committee for Medicinal Products for Human Use (CHMP) when adequate clinical evidence exists on safety and efficacy, but most CUPs are executed on the country level via the local regulatory authority. Only 5 CUPs have been granted through the CHMP so far^34^.

Early access programs are not offered in Kazakhstan, Armenia, Russia, and Ukraine (51)^4^^,^^24^^,^^35^.

France makes extensive use of CUPs for rare diseases, with 70% of the currently reimbursed orphan drugs having had early access before the marketing authorization (59). France is also unique in the fact that it has a legal framework for “early access” for already registered drugs for which a new (medical need) indication is still under assessment, called RTU (Recommendation for Temporary Use) (83, 84). Sixteen products have received an RTU in France so far (83). RTU allows for a more flexible access approach than many other countries, such as the Netherlands, that only allows non-registered drugs for a CUP, regardless of whether the (orphan) indication is approved or not^36^.

In Turkey exist three well-established processes to get access to unapproved drugs, e.g., approved off label-use of registered drugs (e.g., different indication/dosage, or non-approved patient subgroups), Named Patient Imports and CUPs (33, 34). A CUP is acceptable for products that have entered a phase-III clinical program and in case of serious or life-threatening conditions, but only if patients cannot enroll in a clinical trial in Turkey. The Medicines and Medical Devices Agency supervises these programs (33, 34). Scotland has a two-tier program for access to non-routine drugs (i.e., drugs normally not available in the Scottish healthcare system) called PACS, with tier 1 reserved for ultra-orphan drugs and tier 2 for other non-routine drugs (not approved by the Scottish Medicine Consortium). Cost-effectiveness is explicitly excluded from any argumentation for access^37^.

### HTA and Reimbursement Processes for Orphan Drugs

Rare disease populations are small and often show large disease heterogeneity, which leads to difficulties in generating well-powered and controlled randomized clinical trials and useful outcomes. This makes the generation of (high quality) evidence on clinical efficacy and cost-effectiveness troublesome. In turn, HTA assessment processes are usually not tailored to deal with these rare diseases and orphan drugs characteristics. Many countries still reimburse OMP's despite a lower quality of evidence and accept higher prices, often because of societal/compassion-related arguments and the limited total budget impact of the rare disease treatment. Some countries have reduced requirements for evidence and other waivers for rare disease treatments. For example, in France a cost-effectiveness analysis is not required. The Haute Autorité de Santé (HAS) assesses therapeutic benefit, calculated as Service Medical Rendu (SMR), which takes into account: clinical effectiveness, safety of alternatives, clinical relevance in overall treatment strategy, disease severity, population size, and indication (for chronic and preventable diseases) (29).

Similarly, in Turkey orphan drugs are exempt from submitting pharmacoeconomic analyses, which allows OMPs to enter the market faster (if budget impact is within limits) (33, 34). In Romania OMPs receive additional value points (55) during the HTA process, which increases chances for reimbursement (29)^12^.

Since 2012 a conditional reimbursem ent has been possible in the Netherlands, in cases of discussion/doubt over a therapeutic benefit, cost-effectiveness, or the predicted budget impact of a medical intervention (only available for outpatient drugs)^38^. These conditional approvals were intended to ensure patients could get early access to innovative medicines while maintaining budget control. This program came with the requirement to provide additional scientific data within 4 or 7 years (in exceptional cases), for which a subsidy could be requested with a maximum of 400,000 €. However, the number of products that applied for conditional reimbursement up to 2017 turned out to be low. Therefore, the conditional reimbursement program has been replaced by a more general subsidy program, focused at supporting small and medium manufacturers^38^.

Romania also has a conditional reimbursement program, which aims to allow patient access to new drugs quickly, while still keeping a focus on evidence-based medicine and budget control^39^.

In the UK, NICE performs an HTA assessment using incremental cost-effectiveness ratios which are usually implemented by the regions, although a re-assessment or a purely regional HTA can also be done in Scotland, Wales, and Northern-Ireland. The NICE HTA process is based on a threshold level per ICER, with increasing evidence requirements if certain ICER levels are exceeded. Orphan and ultra-orphan drugs can get higher limits^40^^,^^41^^,^^42^^,^^43^^,^^44^^,^^45^^,^^46^.

The Act to Reorganize the Pharmaceuticals' Market in the Statutory Health Insurance System (AMNOG), introduced in Germany in 2011, changed reimbursement of new innovative drugs considerably^31^. Manufacturers are allowed to set prices freely during the first year after marketing authorization, with a mandatory 7% discount to statutory health insurances. An “early benefit assessment” is done after 12 months, after which reimbursement will be recalculated, taking into account the perceived additional benefit of the medicine^47^ (85). Lower evidence thresholds for OMPs were applied within the process and an automatic “additional benefit” for OMPs was assumed, with no necessary comparison against alternative therapies. This streamlined and simplified the reimbursement process for OMPs considerably. The “Legislation for more safety in the supply of pharmaceuticals” (GSAV) introduced in 2019 changed several parameters for OMP reimbursement in Germany, by removing several benefits for OMPs and increasing the likelihood of price reductions for OMPs (67, 86). Under GSAV, OMP manufacturers are more likely to have to invest in data collection activities (e.g., patient registries) and perform comparative analyses. The automatic added benefit clause is removed for OMPs with an annual revenue >€50 M. In this case, a comparative analysis will have to be provided. GSAV now includes both hospital as outpatient costs in the revenue calculations, increasing the likelihood of exceeding the threshold. G-BA will be authorized to perform periodic re-evaluation of the drug's benefits (and conduct price negotiations if deemed necessary). The actual impact of GSAV on orphan drugs, i.e., availability/patient access, pricing, time to market, and disease/drug understanding, remains to be seen. GSAV legislation might lead to more structured, approach toward Real World Evidence (RWE) creation in the rare disease/orphan drug field. It is possible that Germany will push these topics onto the EU agenda during its co-presidency in 2020/2021 (67, 86).

Some countries are looking at novel and alternative methods of assessing orphan drugs, such as Poland who is considering to use MCDA (Multi-criteria decision analysis) in its HTA policies^48^.

A detailed overview of HTA and reimbursement processes is presented in Table 3.

**Table 3:** Comparison of reimbursement systems of orphan drugs and rare diseases policies (15, 16, 29, 70–72)^4^^,^^24^, (68)^30^^,^^31^, (11, 69)^32^, (73–75)^33^, (76–84)^34^^,^^35^^,^^36^^,^^37^^,^^38^^,^^39^^,^^40^^,^^41^^,^^42^^,^^43^^,^^44^^,^^45^^,^^46^^,^^47^, (67, 85, 86)^1^^,^^48^, (87)^2^^,^^3^, (88, 89)^4^^,^^5^, (90, 91)^6^^,^^7^^,^^8^^,^^9^, (92, 93).

**Country**	**Reimbursement/HTA process**
AR	No specific reimbursement process for OMP's. No defined HTA process.
DE	The AMNOG Act requires manufacturers to send in a dossier at the time of regulatory approval (and <1 month after indication change) to the Federal Joint Committee (FJC), the decision-making body of the joint healthcare representatives—HCP's, hospital association, and sickness funds) to demonstrate additional benefit of the drug over a comparator drug. After the additional benefit is granted by the G-BA, a reimbursement price is negotiated between manufacturer and GKV-SV (National Association of Statutory Health Insurance Funds). A budget cap of €50 M per active substance was introduced in 2016. After 12 months, practical benefit was assessed and reimbursement adjusted accordingly. Reimbursement prices that are negotiated on the national level are published. Afterwards, the more than 100 health insurances further negotiate discounts with the manufacturer, which are not publicly available. Except for OMPs, G-BA lets IQWiG (Institute for quality and science in healthcare) assess the proposed additional benefit with the dossier submission at the time of marketing authorization, with five benefit categories: major, considerable, minor, non-quantifiable, no additional benefit. Evidence quality is taken into account, based on the number of studies, evidential certainty and clinical outcomes, resulting in three possible scores: proof, indication or hint of benefit. Four clinical outcomes are measured: mortality, morbidity, adverse events, and health-related quality of life (HRQoL). Patient subgroups can be excluded in case of no added benefit. If projected sales are < €1 M, no full dossier is needed. Until 2019, OMPs with an EMA marketing authorization were viewed as automatically having an established additional benefit over existing therapies (i.e., the 'no additional benefit' score was excluded from OMP benefit scores). After 12 months, an early benefit assessment is performed after which prices can be renegotiated. This changed in 2019, when the GSAV bill introduced a new clause for OMPs that exceed the 50 M annual revenue threshold: in this case drug manufacturers need to perform a comparative analysis with an appropriate comparator drug within 3 months. (Hospital) Inpatient costs are now also to be included in the 50 M budget vs. only outpatient cost before 2019, increasing the likelihood for OMPs to exceed the threshold. Under G-BA can require drug manufacturers to setup data collection programs (patient registry data) according to G-BA rules, as well as require physicians and hospitals to provide OMP administration data to registries in order to be allowed to prescribe these drugs. The costs of these observational data collection activities would have to be covered by drug manufacturers. GSAV authorizes G-BA to perform periodic reassessment of the benefit analysis with new (registry) data. GSAV can lead to an increased number of price-renegotiations/reductions. Arbitration procedures can be started in case of conflicting views between manufacturer and IQWiG/GBA.
FR	No specific reimbursement criteria exist for rare diseases (standard HTA applies), however, a cost-effectiveness analysis is not needed. HAS assesses therapeutic benefit, calculated as Service Medical Rendu (SMR), which takes into account: clinical effectiveness, safety of alternatives, clinical relevance in overall treatment strategy, disease severity, population size, indication (for chronic and preventable diseases). The SMR defines the drug reimbursement level for drugs (three levels exist). The MoH is responsible for final reimbursement. For drugs which HAS considers irreplaceable, reimbursement is set at 100%. HAS also assesses the ASMR indicator (Amelioration du Service Medical Rendu), i.e., therapeutic improvement in comparison to other available treatments and sets the price level based on this value (five possible levels). No specific reimbursement criteria exist for rare diseases. The SMR defines three reimbursement levels for drugs. The MoH is a decision maker. For drugs which HAS considers irreplaceable, reimbursement is set at 100%. HAS also assesses the ASMR indicator (Amelioration du Service Medical Rendu), i.e., therapeutic improvement in comparison to available treatments and sets the price level based on this value (five levels exist). Standard HTA process applies to OMPs, however, a cost-effectiveness analysis is not required. HAS assesses therapeutic benefit, calculated as Service Medical Rendu (SMR), which into account: clinical effectiveness, safety of alternatives, clinical relevance in overall treatment strategy, disease severity, population size, indication (for chronic and preventable diseases).
KAZ	Healthcare is generally funded by the State and is free for all citizens. Treatment of rare diseases is covered within the national healthcare budget, and no special reimbursement rules exist for OMP's. However, OMP funding needs to be applied for by the regions, after which budget is granted by the State, based on individual patient characteristics (e.g., body mass/dosing). OMP's need to be registered in Kazakhstan or elsewhere and be on the official orphan drug list to be eligible for reimbursement. All medical interventions are monitored under supervision of the MoH. No specific HTA process for OMPs.
LV	Drugs listed on the national reimbursement drug list are reimbursed, based although individual patient reimbursement decisions can be made a by the medical council (limit: 14,229 Euro per patient/year). The national reimbursement list has three sections: List A with therapeutically equivalent drugs (generic drugs); List B with drugs without therapeutic equivalent; List C with drugs costing more than 4,269 Euro per patient per year. The manufacturer must reimburse at least 10% of the costs of drugs on list C for a defined number of patients. Other OMP's can be reimbursed on an individual basis in medical need (life-threatening situations) if costs are < €14,229 per year, which is assessed by the National Drug Agency. Co-payment is needed in case of additional costs, by patient or manufacturer. This does not limit access substantially. Between 2008–2011, 300+ patients had successful individual negotiations. Pediatric rare disorders can receive special reimbursement rules. The NHS evaluates therapeutic value, price, expected budget impact and cost-effectiveness for each drug before it is included in the reimbursement list. No specific HTA rules for OMPs. List C decisions are made annually, depending on budget and total budget impact of the treatment.
NL	OMP's go through the same HTA process as all other “specialist drugs,” which are assessed based on the “risk” they pose to the overall Dutch basic insurance coverage, taking into account budget impact, lack of control over the use of the product, doubts on the quality of evidence, etc. If the risk is considered high, a formal HTA assessment is done. A price >€25,000 per patient per year is also defined as a risk factor, however, if total budget impact is small (< €2.5 M per year), ZiN will likely not do an assessment. Due to a frequent lack of evidence for OMP's, the rarity, severity, and societal impact of the disease will be considered. Hospital drugs (mainly specialty care) that either are expected to have a high per patient cost, or a high total budget impact, can be put into a “sluice” (waiting room) by the minister of health. This means a delay in reimbursement until a positive evaluation, restrictions for use have been put in place and/or a successful price negotiation has been done by the MoH (undisclosed).
PL	No special reimbursement rules for OMPs. A reimbursement application is sent to the MoH, which transfers it to AOTMiT for evaluation (containing clinical effectiveness, cost-effectiveness, and budget impact analyses). AOTMiT gathers and assesses information on health, social, economic, and ethical aspects of medicinal technology. The Transparency Council (part of AOTMiT) gives its recommendation on pricing and reimbursement and the final recommendation is issued by the President of AOTMiT. Final approval is given by the Healthcare Minister. Most OMPs are reimbursed within “Drug Programs” (DPs), introduced by the MoH in 2012 for expensive medical technologies replacing previous “therapeutic programs.” DPs are mainly designed to control consumption of the most expensive drugs.
	As a tailored approach to HTA for OMPs does not currently exist in Poland, standard HTA rules for “standard” medicinal products apply, which take into account: health priorities, results of sequelae of disease, public health significance, social preferences, organizational, legal aspects, and ethical aspects. The cost-effectiveness threshold is based on an ICER (Incremental cost-effectiveness ratio) that needs to be lower or equal to 3×GDP per capita to consider a medical technology cost-effective (3 × 41.985 PLN = 125.955 PLN~29.989 EUR in 2016, EUR rate from 16.03.2018 1EUR = 4.2PLN).
RO	OMP's are reimbursed within the National Program for rare disorders and National Program for treatment of chronic disorders (list C2), and provided for free. HTA was introduced in 2014, with separate rules for reimbursement of OMPs. In order to be included in the reimbursement list, medicinal products need to gain a minimum of 60 points (out of 80) during HTA. Results between 60 and 79 ensure conditional reimbursement, with price negotiation and by using risk-sharing tools (agreements on cost-volume, cost-volume-outcome). Drugs with an orphan designation assigned by EMA automatically get 55 points and depending on the reimbursement status in other EU countries points are added: 0 points if the drug is reimbursed in up to 2 EU countries 10 points if the drug is reimbursed in 3–7 EU countries 20 points if the drug is reimbursed in 8–13 EU countries 25 points if the drug is reimbursed in at least 14 EU countries.
RU	The reimbursement system is quite complex, consisting of many lists, programs and levels of reimbursement. OMP's can be reimbursed on federal and regional levels. Federal reimbursement is based on the Vital and Essential Drug List (VEDL)—a list of reimbursed drugs with price limits. Federal benefits are available if rare disease patients belong to one of the “privileged categories” of citizens such as veterans, invalids or victims of the Chernobyl and Mayak disasters. Orphan drugs are mainly reimbursed within two programs, the high-cost Nosologies List and the orphan diseases list. Within the seven nosologies program, funded on the federal level, the treatment for those diseases is reimbursed: hemophilia, cystic fibrosis, pituitary nanism, Gaucher disease, lymphoid malignant neoplasms, hematopoietic and related tissues, multiple sclerosis, hemolytic-uremic syndrome, juvenile arthritis with systemic onset, mucopolysaccharidosis type I, II, and VI. Although the reimbursed treatments on the 24 orphan diseases list are defined on the federal level, funding is done regionally. If budget allows, treatment for other rare disorders (not on one of the lists) can be reimbursed. There is no special HTA for OMPs, the same rules apply as for other medicinal products.
TR	All OMP reimbursement applications are assessed by the Medical and Economic Evaluation Commission, which informs the Reimbursement Commission that will make a final decision. The TITCK, SGK, and the Ministry of Finance are part of the Medical and Economic Evaluation Commission and the Reimbursement Commission. Orphan drugs are exempt from submitting pharmacoeconomic analyses in contrast to other medicinal products, which allows OMP's to enter the market faster (if budget impact is within limits).
UA	In 2016 the new legislation on HTA was implemented. The new regulation introduced criteria (such as morbidity level, disease prevalence, evidence on comparative effectiveness, safety) which are taken into consideration in order to include medicinal products to National essential medicines list (NLEM). In addition a pharmacoeconomic analysis must be provided while applying for the reimbursement. An expert Committee on the Selection and Use of Essential Medicines was appointed by the MoH for decision making concerning the inclusion of medicinal products to NLEM. No specific reimbursement and HTA processes for OMPs exist. In January 2019 HTA Department was established at the State an Expert Centre of the MoH to prepare recommendations and inform decisions on medical technologies financed by the state funds. The main stakeholders are the central government (Cabinet of Ministers), the MoH, the Ministry of Finance and local governments. In 2019 there are 41 national programs that are approved annually for public (state) procurements for diseases, rare diseases in particular, through which OMP's are procured annually via international organizations (UNDP, Crown agents, UNICEF). Currently there are clinical protocols approved by the MoH for treatment of mucopolysaccharidosis, Gaucher disease, epidermolysis bullosa, cystic fibrosis, phenylketonuria, Wilson's disease.
UK	**England** NICE performs HTA assessment using (incremental) cost-effectiveness ratios, with thresholds for medicines (incl. orphan drugs): below the £20,000 limit NHS reimbursement is based mainly on cost-effectiveness data, between £20,000–£30,000 more data is needed e.g., degree of ICER certainty, innovativeness, whether or not the drug is life-extending at end of life, etc. Above £30,000 evidence needs to be stronger. For very rare disorders (1 <50,000) the HST (Highly Specialized Technologies) programme is used, which uses an ICER QALY limit of £100,000. If costs remain below that the assessment will be based on standard cost-effectiveness analysis. Above the limit evidential certainty, the innovation level and actual effectiveness increase (QALY gains) will be taken into account and a QALY modifier can raise the cost limit up to £300,000 per incremental QALY. In 2017 NICE introduced a “budget impact test” with a limit of £20 million (over 3 years), set by the NHS. If the limit is exceeded a commercial negotiation is triggered, special arrangements need to be made and reimbursement can be delayed or phased in over a longer period. Expensive OMP's can also be procured via the Cancer Drug Fund (budget £340 M in 2016), a dedicated budget for innovative costly treatments too expensive for common NHS reimbursement (after NICE recommendation), and also via an Individual Funding Request to the NHS. **Wales** Wales is generally following NICE' reimbursement recommendations, but has its own agency All-Wales Medicines Strategy Group (AWMSG) which can approve drugs for reimbursement. A special treatment fund for high-cost drugs has been introduced in 2017. **Scotland** The Scottish Medicines Consortium (SMC), the Scottish equivalent of NICE, reviews all newly approved medicines, including orphan and ultra-orphan drugs. The HTA process is similar to England, with similar ICER QALY thresholds (£20 and £30 k). The Scottish NHS boards are not obliged to follow SMC's advice. A separate fund exists dedicated to funding expensive medicines, including rare disease treatments, called the New Medicines Fund. Since 2014 manufacturers can ask SMC to convene a Patient and Clinician Engagement (PACE) group, if their drug is not recommended for reimbursement by the New Drug Committee (NDC). PACE was setup after the realization that existing cost-effectiveness thresholds were not always suitable for (ultra)rare diseases and end-of-life conditions. PACE is aimed at enlarging the role of expert physicians and patients in the decision-making process. Orphan drugs for ultra-rare diseases can receive additional flexibility in the process. **Northern Ireland** The Department of Health (DH) in Northern Ireland assesses all NICE recommendations for local implementation. Very rare drugs approved via NICE HST assessment will be approved for reimbursement.

*^1^https://www.gkv-spitzenverband.de/english/statutory_health_insurance/amnog_evaluation_of_new_pharmaceutical/amnog_english.jsp (accessed September 18, 2019). ^2^Interview with French key opinion leader (accessed July 26, 2019). ^3^https://www.has-sante.fr/portail/upload/docs/application/pdf/2014-03/pricing_reimbursement_of_drugs_and_hta_policies_in_france.pdf (accessed September 18, 2019). ^4^https://www.zorginstituutnederland.nl/over-ons/programmas-en-samenwerkingsverbanden/horizonscan-geneesmiddelen/sluis-voor-dure-geneesmiddelen (accessed September 18, 2019). ^5^http://www.korektorzdrowia.pl/wp-content/uploads/3.-wojciech-matusewicz-1.pdf (accessed September 30, 2019)*.

*^6^Order of MOH No. 84 dated 11.02.2016, Order of MoH No. 1050 dated 07.10.2016. Available online at: http://www.apteka.ua/article/362317 (accessed September 30, 2019). ^7^http://www.apteka.ua/article/390509 (accessed September 30, 2019). ^8^Order of MOH No. 778 dated 27.10.2014 http://zakon3.rada.gov.ua/laws/show/160-2015-%D0%BF (accessed September 30, 2019). ^9^https://www.scottishmedicines.org.uk/how-we-decide/pace/ (accessed September 30, 2019)*.

## Discussion

### Limitations of the Study

In order to get a complete overview of the Eurasian region, many more countries would have to be included, however, this went beyond the scope of this article and would overly enlarge it. This overview presents the most recent information that was possible to retrieve at the time of writing, but policies and regulations are continuously changing. Sometimes new information is difficult to find and only available in local languages. The politicization of the (orphan) drug price debate results in shifting political viewpoints highly dynamic healthcare policies. Not all country data is comparable, i.e., mismatches exist in definitions, different aspects of rare disease policies that are covered and the level of detail, on top of structural differences in healthcare systems. To keep this information relevant and up-to-date, research should be done periodically to expand and include the latest information. The German GSAV shows that new and extensive policies can be introduced quickly, especially in an era of rising cost-awareness. Sharing scientific progress and relevant policy developments in a collaborative manner is very relevant in the orphan drug arena, where knowledge and experience are often scarce. A publicly accessible “policy repository” could be a useful tool for researchers and policy makers to share best practices and combine efforts, but which would require continuous input and resources.

This study shows that large differences exist between selected countries with regard to orphan drug policies, solutions, available healthcare budgets, and the level of patient access. This applies to EU vs. non-EU countries, EU member states, and even within a single country. Despite these variations that make it difficult to create a comprehensive overview of policies or generate a clear-cut conclusion, the authors have attempted to capture a representative picture.

### Newborn Screening

Good examples of intra-country differences are newborn screening and orphan drug reimbursement between the regions of the UK (i.e., Northern Ireland vs. Scotland, England, and Wales) and in Moscow vs. the rest of Russia. Newborn children are screened for the highest number of rare disorders in Poland (28), followed by The Netherlands (20). On the lower end of the scale, Kazakhstan, Latvia, Romania screen for only two diseases. Russia has the region with the broadest newborn screening in this review (35 RD's in Moscow), although large parts of the country have a much smaller program^9^. Aggregation of data concerning newborn screening is not always straightforward, since many rare metabolic disorders have different names or subtypes which can be considered either as one disease or as separate rare conditions, depending on publications and local guidelines. Disease carriership is sometimes counted as a separate condition (e.g., sickle cell disease and sickle cell carriership in the Netherlands). Overall though, the national plans have led to expansion of the amount of screened diseases. Implementing a new screened disease requires testing and validation of new technology, so the implementation status is sometimes not clear to the public.

Despite the wide international consensus on the efficiency of NBS for phenylketonuria in terms of costs and effectiveness, this consensus is challenged as new disorders are proposed to be included in a NBS program (93). NBS programs might be relatively inexpensive, even when the confirmatory diagnostic tests for both the true and false positives and the follow-up and treatment costs of affected children are included. However, the high heterogeneity of the disorders potentially detected by screening, and the lack of robust and long-term scientific evidence on the effectiveness of the treatments and the natural history of the disorders, pose a number of methodological difficulties that limit the applicability of standard pharmacoeconomic evaluation methods to prove its cost-effectiveness.

### Disease Registries, National Plans for Rare Diseases

The national plans have stimulated the creation of registries as scientific centers, but implementation varies per region. Government publications have been reviewed to assess the availability of patient/disease registries, but whether the mentioned registries are operational, being implemented or merely announced is sometimes not transparent. Reorganization, grouping, and renaming of registries is common. Other institutions, such as universities or patient organizations are often involved in gathering this data but they were not included in this review. The ongoing implementation of national plans in the EU since 2013 has reinforced the international recognition of rare disorders in governmental programs substantially, leading to alteration and implementation of various policies. The newly approved European Reference Networks are a good example. The results of this increased data gathering will hopefully lead to better understanding, diagnosis and treatment of rare diseases, but this will take time.

### Access to Treatment

The main effect of the fragmentation of reimbursement policies is unequal access to treatment. The number of reimbursed OMPs in the selected countries ranges from 100+ OMPs in The Netherlands, Germany, France to zero in Armenia. The EU countries are leading in access to OMPs but positive developments for patients are also seen outside the EU, e.g., in Russia and Kazakhstan. Like France, Turkey also has implemented regulatory flexibility, by allowing the use and importation of drugs for non-registered orphan indications (i.e., managed off-label use).

In some countries legislation is completely lacking, leaving patients without many options to get access to any (expensive) medication, such as in Armenia. Early access programs can temporarily alleviate an urgent medical need for OMP with a low burden for society and patients, and since these are relatively easy to implement they should be introduced in all countries.

### HTA and Reimbursement

No specifically tailored HTA approaches were identified for orphan drugs, although waivers and reduced data requirements are often present in some form or another. Many countries use standard HTA processes but do reimburse OMP's despite lacking evidence.

Rare diseases commonly place a large burden on family and caregivers, the impact of which is usually not taken into consideration in standard cost-effectiveness analyses (94–96). In light of the lack of appropriate HTA tools that can incorporate benefits and costs specific to rare disease treatments beyond the standard cost per QALY, e.g., socio-economic aspects, Multi-Criteria Decision Analysis (MCDA) is an approach that could be considered. MCDA can support decision-making processes by capturing and weighting a range of factors of a certain intervention, the result of which is one composite outcome score. This outcome can be used for comparison between technologies (97, 98). MCDA has been implemented in legislation in Lombardia (for diagnostics, medical devices, interventional procedures, and medicinal products including OMPs) and also in Hungary for new hospital medical technologies (99, 100). Poland is currently considering the use of MCDA for this purpose.

Researchers in the rare disease area are also looking into the use of MCDA, which has resulted in a list of scientific publications and MCDA model designs, but full consensus on MCDA is still lacking and further research is needed to support implementation in (rare disease) HTA (94, 101–116).

Reimbursement rules are harder to unify than regulatory legislation, due to regional economic and political differences, also in the EU. However, signs of international cooperation are visible, as the European Parliament Committee on Environment, Public Health and Food Safety (ENVI) is investigating shared HTA and pricing projects in the EU^49^. The European Mechanism of Coordinated Access to Orphan Medicinal Products (MoCA) project is a step toward international harmonization and improvement of patient access to OMPs. This platform aims to facilitate an early dialogue on pricing and reimbursement already during the development phase of OMPs between pharmaceutical companies and competent authorities^50^ (117). The Transparent Value Framework (TVF) which is an MCDA-like method developed by Hughes-Wilson, was also tested within this project in order to develop a coordinated mechanism between the 12 participating Member States and orphan drug developers to evaluate the value of OMPs^51^.

The EU HTA Regulation that was announced builds on these earlier initiatives, centered around the concept of a centrally performed Joint Clinical Assessment (JCA) that can be used by national HTA agencies (8). Economic factors will probably still be evaluated nationally, but a central “clinical value” assessment would avoid duplication efforts, reduce workload and make the HTA process more transparent and predictable for all stakeholders. Given the pressure on costs, especially in the area of expensive medicines, sharing, and implementation of new cost-reduction policies is to be expected.

### New Scientific Methodology

Several new scientific and methodological approaches are being developed to improve evidence generation and analysis for small population groups, including new trial designs and clinical endpoints such as was done in the EU FP7 framework recently and its subprograms IDEAL (Integrated DEsign and AnaLysis of small population group trials), InSPiRe (Innovative methodology for Small Populations Research) and ASTERIX (Advances in Small Trials dEsign for Regulatory Innovation and eXcellence)^52^. Goal Attainment Scaling (GAS) came out as an example of a “rediscovered” endpoint that can capture individual and heterogeneous symptoms via personalized outcome parameters (118, 119). *N* = 1 trial methodology (single-subject design) allows to perform a double-blind randomized placebo-controlled trial with one single patient, via randomized treatment cycles of both drug and control. Although limitations exist (e.g., suitable for chronic conditions only), the method seems appropriate for ultra-rare diseases (120). Drug manufacturers can benefit from all these developments, e.g., with improved clinical methodology for rare diseases and clear, predictable and transparent orphan drug legislation and HTA processes that are adapted for orphan drugs. In turn, this can support regulators and payers when assessing the value and benefits of OMPs. It is not clear, however, if and how fast these new developments will result in actual benefits for rare disease patients, i.e., improved access to a wider range of drugs. International medical and scientific collaboration for rare diseases already exists for a while (e.g., Orphanet, Eurordis), but cooperation on HTA issues and patient access is still lagging behind. Unified international approaches to tackle common issues surrounding orphan drugs are being developed slowly.

This article has looked at a broad range of initiatives over a wider region, and it can be concluded that no single country in this review can be marked as having the “most optimal” rare disease solutions. A broad national newborn screening program can be accompanied by a relatively small reimbursement program in the same country. Learnings should be taken from the respective national experiences and by sharing of policy related information, which was also the aim of this publication. In order to create additional momentum, initiatives that can effectively support orphan drug access should be prioritized and placed on the public agenda, preferably supported by strong political entities. The rarity and complexity of the rare disease/orphan drug arena makes collaboration and harmonization essential. Only in this way the 350 million people suffering from rare disorders around the world can hope to expect fair and equal access to treatments in the future^53^. Continuous research and sharing of information is highly recommended to identify and promote best practices in the rare disease policy field.

## Author Contributions

MC coordinated the group of researchers in gathering and unifying information and provided information about Poland. AB-K performed data analysis, data gathering, wrote the publication, and provided information about Poland, Latvia, France, United Kingdom. KA provided information about Turkey. MD editorial changes and provided information about Armenia. KG provided information about Kazakhstan. MH-V provided information about Russia. AT-S provided information about Romania. CK proofread and provided information about The Netherlands and Germany. OP and OZ provided information about Ukraine. NK provided information about Armenia. JS-C provided information about Poland.

### Conflict of Interest

The authors declare that the research was conducted in the absence of any commercial or financial relationships that could be construed as a potential conflict of interest.

## Footnotes

^1^https://www.eurordis.org/sites/default/files/publications/Fact_Sheet_RD.pdf (accessed September 18, 2019).

^2^http://www.ema.europa.eu/docs/en_GB/document_library/Leaflet/2017/12/WC500240710.pdf (accessed September 18, 2019).

^3^https://www.england.nhs.uk/commissioning/spec-services/highly-spec-services/ (accessed September 18, 2019).

^4^Interview with Armenian key opinion leader (accessed September 5, 2019).

^5^https://www.nza.nl/regelgeving/beleidsregels/BR_CU_2018__Weesgeneesmiddelen (accessed September 18, 2019).

^6^https://news.gov.scot/news/treatments-for-rare-conditions (accessed September 18, 2019).

^7^https://www.g-ba.de/informationen/richtlinien/15/ (accessed September 18, 2019).

^8^https://muko.info/ueber-mukoviszidose/neugeborenen-screening.html (accessed September 18, 2019).

^9^https://mosgorzdrav.ru/ru-RU/news/default/card-print/1802.html (accessed September 18, 2019).

^10^https://dosyaism.saglik.gov.tr/Eklenti/11173,259822214447pdf.pdf?0 (accessed September 18, 2019).

^11^https://newjournal.ssmu.kz/publication/249/realizatsiya-skriningovykh-programm-vkazakhstane-na-sovremennom-etape/ (accessed September 18, 2019).

^12^Order of Romanian MoH no. 387/2015 regarding the change and completion of the Order of the MoH no. 861/2014 for approving the criteria and methodology for health technology assessment, Ordinul nr. 387/2015 privind modificarea şi completarea Ordinului ministrului sănătăţii nr. 861/2014 pentru aprobarea criteriilor şi metodologiei de evaluare a tehnologiilor medicale.

^13^http://www.nhs.uk/Conditions/pregnancy-and-baby/Pages/newborn-blood-spot-test.aspx (accessed September 18, 2019).

^14^http://www.gov.scot/Topics/Health/Services/Screening/Newborn (accessed September 18, 2019).

^15^https://ec.europa.eu/health/rare_diseases/national_plans/detailed_en (accessed September 18, 2019).

^16^http://www.europlanproject.eu/NationalPlans?idMap=1 (accessed September 18, 2019).

^17^Dutch National Plan Rare Diseases. http://www.nfu.nl/img/pdf/nationaal-plan-zeldzame-ziekten.pdf (accessed September 18, 2019).

^18^https://www.bundesgesundheitsministerium.de/fileadmin/Dateien/3_Downloads/N/NAMSE/National_Plan_of_Action.pdf (accessed September 18, 2019).

^19^https://assets.publishing.service.gov.uk/government/uploads/system/uploads/attachment_data/file/684461/Rare_Disease_Policy_Board_-_Second_Progress_Report_2016-2018.pdf (accessed September 18, 2019).

^20^https://www.england.nhs.uk/wp-content/uploads/2018/01/implementation-plan-uk-strategy-for-rare-diseases.pdf (accessed September 18, 2019).

^21^Interview with Romanian key opinion leader (accessed August 11, 2019).

^22^Interview with Polish and Romanian key opinion leader (accessed August 13, 2019).

^23^https://bip.kprm.gov.pl/kpr/bip-rady-ministrow/prace-legislacyjne-rm-i/prace-legislacyjne-rady/wykaz-prac-legislacyjny/r32597703041012,Narodowy-Plan-dla-Chorob-Rzadkich.html (accessed September 18, 2019).

^24^Interview with Russian key opinion leader (accessed September 1, 2019).

^25^https://www.orpha.net/orphacom/cahiers/docs/GB/Registries.pdf (accessed September 18, 2019).

^26^http://www.rejestrwad.pl/o-rejestrze/historia-prwwr (accessed September 18, 2019).

^27^European Reference Networks. https://ec.europa.eu/health/ern/networks_en (accessed September 18, 2019).

^28^http://www.anm.ro/_/ORDINE/ORDIN%20%20%20Nr%20387_2015_modif%20si%20complet%20OMS%20861_2014.pdf (accessed September 18, 2019).

^29^https://www.zakon.kz/4777791-nacionalnyjj-reestr-redkikh.html (accessed September 18, 2019).

^30^https://www.slideshare.net/OHENews/access-to-orphan-drugs-in-the-uk-and-other-european-countries (accessed September 18, 2019).

^31^http://skc-beratung.de/wp-content/uploads/2017/03/White_Paper_SKC.pdf (accessed September 18, 2019).

^32^https://www.cnas.ro/page/listamedicamentelor-2019.html (accessed September 18, 2019).

^33^http://www.iokpb1.ru/perechen-7-nozologii-2019.pdf (accessed September 18, 2019).

^34^http://www.ema.europa.eu/ema/index.jsp?curl=pages/regulation/general/general_content_000293.jsp&mid=WC0b01ac05809f843c (accessed September 18, 2019).

^35^Interview with Ukrainian key opinion leader (accessed September 5, 2019).

^36^https://www.cbg-meb.nl/mensen/voor-handelsvergunninghouders/inhoud/voor-aanvraag-handelsvergunning/compassionate-use-programma (accessed September 18, 2019).

^37^https://www.gov.scot/news/reforming-access-to-new-medicines/ (accessed September 18, 2019).

^38^Letter of the Healthcare Minister on restructuring of the conditional reimbursement ruling https://www.rijksoverheid.nl/documenten/kamerstukken/2017/02/21/kamerbrief-over-herinrichting-van-de-regeling-voor-voorwaardelijke-pakkettoelating (accessed September 18, 2019).

^39^http://www.cnas.ro/casbr/page/contracte-cost-volum-cost-volum-rezultat.html (accessed September 18, 2019).

^40^https://www.england.nhs.uk/wp-content/uploads/2013/04/cdf-sop.pdf (accessed September 18, 2019).

^41^http://gov.wales/newsroom/health-and-social-services/2017/170110fund/?lang=en (accessed September 18, 2019)

^42^https://www.scottishmedicines.org.uk/About_SMC/Policy_statements/A_Guide_to_Quality_Adjusted_Life_Years (accessed September 18, 2019).

^43^http://www.bbc.com/news/uk-scotland-scotland-politics-32761132 (accessed September 18, 2019).

^44^https://www.nice.org.uk/Media/Default/About/what-we-do/NICE-guidance/NICE-highly-specialised-technologies-guidance/HST-interim-methods-process-guide-may-17.pdf (accessed September 18, 2019).

^45^https://www.nice.org.uk/process/pmg9/chapter/the-appraisal-of-the-evidence-and-structured-decision-making (accessed September 18, 2019).

^46^The interview with British key opinion leader (accessed July 14, 2019).

^47^The interview with German key opinion leader (July 5, 2019).

^48^https://www.gov.pl/web/zdrowie/rada-ministrow-przyjela-dokument-polityka-lekowa-panstwa-20182022 (accessed September 18, 2019).

^49^http://www.europarl.europa.eu/RegData/etudes/STUD/2015/542219/IPOL_STU(2015)542219_EN.pdf (accessed September 30, 2019).

^50^https://www.eurordis.org/content/moca (accessed September 30, 2019).

^51^Hughes-Wilson W. *MoCA Concept and Pilot Project, Feedback From the Process Around the First Pilot Project*. Berlin: ECRD. Available online at: http://download2.eurordis.org.s3.amazonaws.com/moca/presentations/PRES-2014-05%20MoCA%20Concept%20and%20Pilot%20Project%20(Hughes-Wilson).pdf (accessed September 30, 2019).

^52^https://ec.europa.eu/research/fp7/index_en.cfm (accessed September 30, 2019).

^53^https://globalgenes.org/rare-diseases-facts-statistics/ (accessed September 30, 2019).
